# THE GEOGRAPHY OF FAMILY CAREGIVING IN AN AGING SOCIETY: WHO MOVES CLOSER AND WHY

**DOI:** 10.1093/geroni/igad104.3072

**Published:** 2023-12-21

**Authors:** Stipica Mudrazija, Elizabeth Peters, Fernando Hernandez Lepe

**Affiliations:** University of Washington, Seattle, Washington, United States; Urban Institute, Washington, District of Columbia, United States; Urban Institute, Washington, District of Columbia, United States

## Abstract

An emerging literature examines the dynamic aspect of the link between parental health and parents-adult children geographic distance, yet who moves when a parent needs care—the parent or the child—and why remains unexplored. Using pooled 2004-2014 data from the Health and Retirement Study on respondents age 65 years or older and their adult children and detailed geographic information, we analyze who moves when parents and children move in together or move closer without coresiding, and who benefits from such moves. Preliminary results reveal that children are roughly four times more likely to move in with parents than parents move in with children, and moving primarily benefits those who move: 89.2 percent of parents who moved reported that either they were the sole beneficiary of the move or that both they and their children benefited, and the same was true of 92.8 percent of children movers. However, as parents’ health declines, they increasingly benefit from the proximity-enhancing moves regardless of who moves. For example, about half of all children who move in with parents following the onset of parents’ health decline reports doing was to benefit their parents, either exclusively of in conjunction with them. Next, we will fit regression models of who moves for newly coresident dyads and dyads moving closer, respectively, as well as models of who benefited from such moves. This research provides new policy-relevant insights because the caregiver supports needed could be different depending on whether it was the parent or the child that moved.

